# Structural brain network in relation to language in school-aged extremely preterm children: A diffusion tensor imaging study

**DOI:** 10.1016/j.nicl.2025.103782

**Published:** 2025-04-12

**Authors:** M. Boumeester, E. Blom, T. Boerma, F. Lammertink, M.P. van den Heuvel, J. Dudink, M.J.N.L. Benders, E. Roze

**Affiliations:** a Department of Pediatrics, Division of Neonatology, Wilhelmina Children’s Hospital, University Medical Center Utrecht, Utrecht, the Netherlands; b Department of Development and Education of youth in Diverse Societies (DEEDS), Utrecht University, Utrecht, the Netherlands; c Institute for Language Sciences, Department of Languages, Literature and Communication, Utrecht University, Utrecht, the Netherlands; d Department of Complex Trait Genetics, Center for Neurogenomics and Cognitive Research, Vrije Universiteit Amsterdam, Amsterdam Neuroscience, Amsterdam, the Netherlands; e Department of Neonatal and Pediatric Intensive Care, Division of Neonatology, Erasmus University Medical Center-Sophia Children’s Hospital, Rotterdam, the Netherlands

**Keywords:** Prematurity, Language development, Diffusion weighted imaging, Connectivity, Network based statistics

## Abstract

Between 22 and 45 % of children born preterm experience difficulties with expressive and receptive language when they reach school age. Little is currently known about the neural mechanisms behind their linguistic performance. This study investigates the brain areas and white matter connections that form the structural language network in extremely preterm-born children who have reached school age. Structural brain connectivity was quantified using diffusion-weighted imaging (DWI) and tractography in *n* = 58 (62 % female) extremely preterm-born children aged 8–12 years. Language outcomes were assessed using the CELF-4-NL Recalling Sentences subtest. Language scores were below average in *n* = 13 (22 %) children. Language outcomes related significantly to a subnetwork of 16 brain regions (*p* = 0.012). The network comprised brain regions from the left hemisphere including the pars orbitalis, middle and superior frontal gyrus, frontal pole, pre- and postcentral gyrus, superior temporal gyrus, insula, caudate nucleus, thalamus, and putamen. In the right hemisphere, the anterior cingulate was part of the network. These findings suggest that extremely preterm children rely mostly on their left hemisphere during language processing, which is similar to typically developing children. However, they seem to use compensatory neural pathways that include brain areas right next to the areas typically involved in language processing. These areas include the pars orbitalis (adjacent to Broca’s area) and the putamen and caudate nucleus (adjacent to the limbic system). It is important to note that language difficulties were not necessarily related to brain injury around birth.

## Highlights

• A structural brain network was identified that relates to complex language in preterm born children at school age.
• This language network contains areas from the known dorsal and ventral language pathways but also other, novel brain regions.
• Language outcomes in preterm children were related to SES, but not to neonatal brain injury.
• Extremely preterm born children might use compensatory neural pathways for language to make up for neuronal dysmaturation.

## 1 Introduction

Infants born extremely preterm (EPT, gestational age < 28 weeks) are at increased risk for long-term neurocognitive morbidities such as verbal language difficulties. These language difficulties have been associated with alterations in white and gray matter structures in the brain, possibly as a consequence of disruptions in brain maturation and mediated by risk factors such as brain injury, inflammation, and infection (De Kieviet et al., 2012, Ment et al., 2009, Stipdonk et al., 2018, Vohr, 2014). Some previous studies examined language in relation to brain microstructure in EPT children using diffusion-weighted imaging (DWI) and tractography (Constable et al., 2008, Feldman et al., 2012, Kallankari et al., 2023, Mullen et al., 2011, Mürner-Lavanchy et al., 2018, Northam et al., 2012, Skranes et al., 2007, Stipdonk et al., 2018). However, the majority were region of interest-based studies, investigating fractional anisotropy (FA) of white matter tracts known to be relevant for language in typically developing children and adults. What makes the present study novel, is that it takes a network-based and data-driven approach to further determine which brain areas and white matter connections make up the language network of EPT school-aged children, acknowledging that language is formed in distributed large-scale networks.

Studies report both receptive (i.e. understanding of information) and expressive (i.e. talking) language difficulties in 20–45 % of school-aged children born preterm compared to term-born peers (e.g. Barre et al., 2011, Luu et al., 2009, Magill-Evans et al., 2002, Nguyen et al., 2018, Northam et al., 2012, Reidy et al., 2013, Stipdonk et al., 2020, Zimmerman, 2018). This is alarming, as well-developed language skills are essential for everyday communication, socioemotional development, and academic achievements (Durkin and Conti-Ramsden, 2007, Johnson et al., 2011, Rose et al., 2018, Twilhaar et al., 2018, Ziegenfusz et al., 2022).

Language difficulties were found in studies using relatively simple language tasks focused on vocabulary or short main clauses but seem most noticeable in assessments of complex language such as the production of a narrative or sentences consisting of main and subordinate clauses (e.g. Crosbie et al., 2011, Lee et al., 2011, Luu et al., 2009, Stipdonk et al., 2020; Van Noort-Van der Spek et al., 2012, Vohr, 2014). Complex language tasks require higher-order cognition to integrate multiple language components such as semantics (i.e., meaning), syntax (i.e., structure) and pragmatics (i.e., language use in context). Difficulties with complex language were found to worsen in preterm born children from 3 to 12 years old (Van Noort-Van der Spek et al., 2012).

Language processing involves a complex neural network, including multiple cortical and subcortical brain areas and white matter connections (e.g. Fedorenko and Thompson-Schill, 2014, Tremblay and Dick, 2016). Growing evidence supports the dual-stream model for language processing (Hickok and Poeppel, 2004, Hickok and Poeppel, 2007). This model proposes a dorsal stream (i.e. superior longitudinal fasciculus and the arcuate fasciculus), involved in phonological processing, and a ventral stream (i.e. inferior fronto-occipital fasciculus), involved in semantic processing, in the language-dominant hemisphere (e.g. Axer et al., 2013, Dick et al., 2014, Fujii et al., 2016, Tremblay and Dick, 2016, Turken and Dronkers, 2011). In addition, some studies indicate that subcortical structures such as the corpus callosum (e.g. Bartha-Doering et al., 2021), thalamus (e.g. Fritsch et al., 2022, Klostermann et al., 2013) and the cerebellum (Mariën et al., 2014, Stipdonk et al., 2021, Vias and Dick, 2017) are part of the language network. The current study examines the association between complex language and diffusion imaging and tractography quantifying whole-brain connectivity. A secondary aim is to determine the relation between neonatal brain injury around birth and language performance at school age.

## 2 Methods

### 2.1 Participants

This study included EPT-born children that were admitted between 2007–2012 to the Neonatal Intensive Care Unit (NICU) of the Wilhelmina Children’s Hospital Utrecht, The Netherlands. They participated in the longitudinal BIOS study: Biomarkers for Outcome at School age in preterm born children (Van der Zwart et al., 2024). At 8–12 years these children were invited to the outpatient clinic to assess neuropsychological functions, perform a brain MRI scan, and collect body materials (not part of this current study). Children were excluded if they had major chromosomal and/or congenital anomalies or were unable to comply with the study procedure. The BIOS study was ethically approved by the medical ethical committee of the University Medical Center Utrecht (protocol NL59105.041.17).

During the BIOS study period, 95 children were included in the study. Fourteen out of 95 children did not participate in the MRI scanning procedure due to no consent of parent(s), the child did not want to participate, or logistic reasons (i.e., COVID-19 restrictions). In addition, 6 children were excluded from the current study due to motion artifacts on the MRI. For 58 out of the remaining 75 children, language was assessed either during the outpatient clinic visit or in a second home visit in case children were fatigued or inattentive to complete the full test battery on the first day.

Characteristics of the included children (*N* = 58, *M*_age_ = 9.1 y_,_ *SD*_age_ = 0.8 y) are presented in Table 1. There was a slight preponderance of females in the study group (*n* = 36 females, *n* = 22 males).

**Table 1:** Patient characteristics.

**Characteristics**	**Study sample (*N* = 58)**
Gender (female/male)	36/22
Gestational age in weeks, *M* (*SD*)	26.6w (1)
Birth weight in grams, *M* (*SD*)	902 g (170)
Singleton/multiple birth	58/0
Kidokoro global score of neonatal brain injury (normal/mild/moderate/severe)	20/28/5/2
Age at scan in years, *M* (*SD*)	9.1y (0.8)
Age difference scan and language test in years, *M* (min, max; *SD*)	0.3y (−2, 3.17; 1.25)
Handedness (left/right)	17/41
Socioeconomic status (low/middle/high)	3/25/30
Speech therapy in past (yes/no)	35/17
Dutch only language at home (yes/no)	49/9

The majority of the children had the scanning session and language test around the same date (*n* = 40). For *n* = 18 children there was ∼ 1 year between language assessment and MRI scanning due to COVID-19.

Neonatal brain injury was assessed on neonatal MRI at 40 weeks postmenstrual age using the Kidokoro global brain abnormality score (Kidokoro et al., 2013). This score is a sum of abnormal brain development in cerebral white matter, cortical and deep gray matter, and cerebellum. Neonatal MRI was available for 55 children. The Kidokoro score was normal or mildly abnormal in the majority of children (87 %). Only 5 children (9 %) had moderate, and 2 (4 %) had severe neonatal brain abnormality.

Socio-economic status (SES) was based on either maternal education level or occupation that was converted to education level using the Dutch National Classification of Occupations ROA-CBS 2014 (BRC 2014) (Statistics Netherlands, 2014) which is derived from the International Classification of Occupations (ISCO-08) (International Labour Organization, 2012). SES was divided on a 3-point scale into low, middle, or high SES. However, because there were only 3 children with low SES, we grouped the low and middle SES groups for further analyses.

9 children grew up in a bilingual household, with another language next to Dutch.

### 2.2 Magnetic resonance imaging

Imaging was performed on a 3 T Philips scanner (Philips Healthcare, Best, The Netherlands), using a 32-channel head coil. For the current study, we acquired 3D-structural anatomical T1 and diffusion-weighted images. Sagittal T1 images were acquired with an echo time of 4.6 ms, inversion time of 947.27 ms, repetition time of 10 ms, flip angle of 8°, voxel resolution of 0.79x0.79x0.80 mm and field of view of 240x240x160 mm. Diffusion-weighted images were acquired with an echo time of 99 ms, repetition time of 3500 ms, 2 mm isotropic voxel resolution, number of directions 105, b-value of 1000, flip angle of 90°and field of view of 224x224x132 mm.

### 2.3 Data processing

#### 2.3.1 DWI tractography

The acquired T1-weighted and diffusion tensor images were preprocessed for tractography analysis (De Lange et al., 2023, Lammertink et al., 2022). We used the anatomical T1 sequence for the preprocessing. FreeSurfer (Fischl, 2012) was used to parcellate the anatomical T1-weighted images into 82 cortical regions using the FreeSurfer’s Desikan-Killiany atlas (Desikan et al., 2006). The individual parcellation maps were then co-registered to the diffusion tensor images. Diffusion tensor images were corrected for eddy current distortions and head motion using FSL EDDY (Andersson & Sotiropoulos, 2016). Next, a FA whole-brain map was derived by estimating the main diffusion direction in each voxel using a tensor fitting algorithm (Alexander et al., 2007, Chang et al., 2012). Finally, DTI-tractography was performed using fiber assignment by continuous tracking (FACT) (Mori et al., 1999), which is deterministic tractography. Reconstruction of white matter streamlines started from eight seeds in every white matter voxel and tracking was discontinued when a streamline showed high curvature (>45°), exited the brain mask or when a streamline entered a voxel with low FA (<0.1). The mean FA value of a streamline was computed as the weighted average FA value over all voxels that a streamline passed.

#### 2.3.2 Network reconstruction

All reconstructed white matter streamlines were combined with the parcellated gray matter maps into individual brain networks. The 82 gray matter regions functioned as nodes in the network. Two areas were considered connected if at least one streamline touched both regions. The weight of each connection was determined by the mean FA of streamlines involved. To control for false positives, a prevalence threshold was applied, such that connections present in at least 50 % of the participants were retained. Results of later analyses were validated using different prevalence thresholds (50–90 %, steps of 10 %).

### 2.4 Linguistic assessment

Language was assessed using the Recalling Sentences subtest of the Dutch adaptation of the standardized Clinical Evaluation of Language Fundamentals − Fourth Edition (CELF-4-NL, Semel et al., 2010). During this test, children repeat sentences of increasing length and complexity as orally presented by an examiner. The sentence repetition test examines linguistic performance at the levels of syntax, morphology, semantics, and lexical knowledge, and can therefore be considered a complex language task (e.g. Van Witteloostuijn et al., 2021). The test was administered according to the official manual of the CELF-4-NL. This means that children aged 8 years started at the first sentence, while children aged 9 years and older started the test from the ninth sentence. The audio was recorded and the task was scored offline by a native Dutch speaker. Each repeated sentence was scored 3 (0 errors), 2 (1 error), 1 (1 or 2 errors) or 0 points (4 or more errors). Testing was terminated after five consecutive null scores. For nine children the test was accidentally terminated too early. For these children, the scores of the sentences that were inadvertently not administered were multiply imputed using the R-package MICE (Buuren & Groothuis-Oudshoorn, 2011). The MICE imputation method is a peer-reviewed, well-documented method for data imputation. The data are ‘missing at random’ due to an administration error by the tester. When data are missing at random, up to 20 % of imputation can be accepted (Van Buuren & Groothuis-Oudshoorn, 2011).

Predictors were the scores of sentences with a minimum correlation of ρ = 0.30 with the target. We ran the imputation algorithm ten times, which resulted in ten imputed datasets. Children’s total score was converted to an age-corrected norm score between 1 and 19. The test has a mean norm score of 10 and a standard deviation of 3.

### 2.5 Statistical analyses

Statistical analyses were performed using R-Studio version 4.0.3 and IBM SPSS Statistics software version 26.0. We examined relations between language data and demographic data such as gender, handedness, SES, and multilingualism at home using *t-*tests. Spearman correlations were used to relate language data to neonatal brain abnormality. All analyses were applied to the ten imputed datasets and then pooled using the MICE pool scalar function applying Rubin’s Rules (Rubin, 1987). An example script for the R code used for data imputation and multiple imputation pooling is added as *supplementary material 1*.

We used network-based statistics (NBS) (Zalesky et al., 2010) to identify interconnected subnetworks that were significantly related to sentence repetition norm scores. NBS is a permutation-based method that identifies subnetworks of edge-wise effects. It gains power by exploiting the extent to which the connections comprising the effect of interest are interconnected. Based on previous literature and data relations, we created an NBS model adjusting for gender, SES, age at scan and age difference between MRI-scan and language test. This model was applied to non-zero Ni × Nj connections of the individual brain networks. The N × N matrix of *F*-statistics and matching *p*-values associated with the relation between FA and language scores was thresholded at a *p*-value of *p* < 0.05 to construct a set of supra-thresholded links. NBS then identified the largest connected component of the thresholded network and assessed its significance using permutation testing by 10.000 times comparing its size against the distribution of greatest component sizes in null-models of permuting subject labels. A *p*-value was assigned to the identified component based on the percentage of the permutations in which the greatest null-model component was larger or equal to the identified component.

NBS was applied to each of the ten imputed datasets. *P*-values of the ten biggest, corresponding components were pooled by z-transformation using the method proposed by Licht (2010). As each imputed dataset resulted in a slightly different biggest component (i.e., differing in size and edges involved), we checked which connections were present in at least 8 of these components to determine the final component. To gain more insight into these analyses, an example of a script for the network-based statistics is provided in *supplementary material 2*.

## 3 Results

### 3.1 Language outcome

The multiply imputed mean norm score of the Recalling Sentences test was 8.4 (*SDpooled* = 3; range 1–17). The imputed mean language norm score was below average (i.e. score < 7) for 13 children (22 %). Language norm scores were related to SES, with children from the high SES group scoring an imputed mean norm score of 9.2 (*SDpooled* = 3) and the children from the low and middle SES group scoring an imputed mean norm score of 7.5 (*SDpooled* = 2.72) (*t*(53.90) = -2.33, *p* = 0.024, *d* = 0.62).

Norm scores were not related to gender (*t*(54.46) = 0.66, *p* = 0.510; *d* = 0.19), left or right handedness (*t*(53.17) = -0.49, *p* = 0.627; *d* = 0.14), bilingual household (*t*(53.61) = -0.16, *p* = 0.870, *d* = 0.07), and the Kidokoro neonatal brain abnormality score (*r* = -0.17, *p* = 0.230).

### 3.2 Structural brain network analyses

**Fig. 1:**
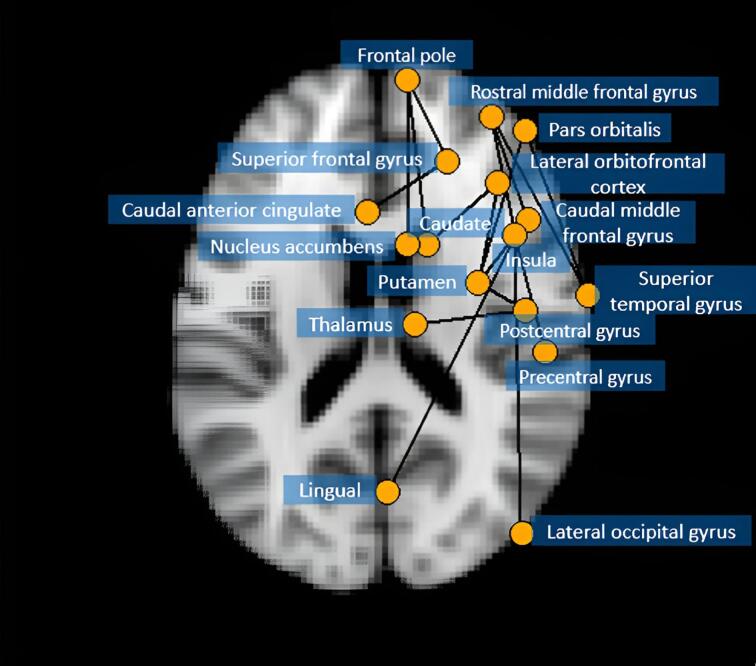
The NBS model projected onto a transverse MRI plane. The results show the network in which FA values were related to language performance.

**Fig. 2:**
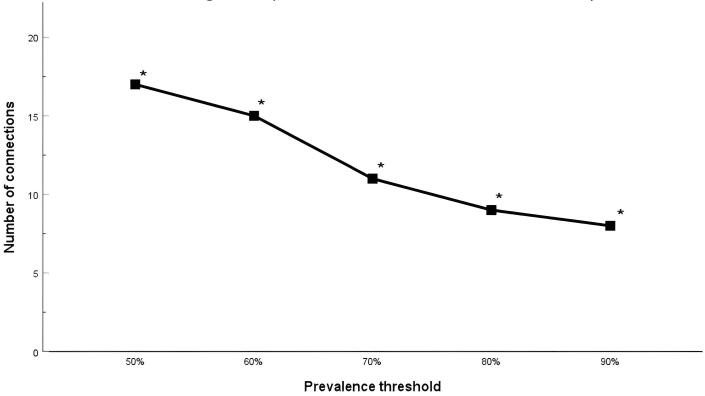
Robustness of the left hemisphere network identified by NBS across prevalence thresholds (50 %: p = 0.012; 60 %: p = 0.040; 70 %: p = 0.018; 80 %: p = 0.026; 90 %: p = 0.027).

The NBS model was adjusted for gender, SES, age at MRI scan, and age difference between MRI scan and language test, and ran for each of the ten imputed data sets to identify neural subnetworks related to sentence repetition norm scores. After pooling *p*-values, one network was identified that related significantly to language outcomes (Recalling Sentences score, *p* = 0.012). This network contains 17 connections that were present in the biggest, corresponding components of at least 8 out of 10 imputed data sets (Table 2). The network consists of 16 brain regions in the left hemisphere including the pars orbitalis, middle and superior frontal gyrus, frontal pole, lateral orbitofrontal cortex, pre- and postcentral gyrus, superior temporal gyrus, insula, caudate nucleus, thalamus, and putamen. In the right hemisphere, the anterior cingulate was part of the network (Fig. 1). The network gradually reduced in size but remained significant across prevalence thresholds of 60 %, 70 %, 80 %, and 90 % (Fig. 2).

**Table 2:** Connections found in the largest significant component (prevalence threshold of 50%) in at least 8 out of 10 imputed datasets.

**Starting node**	**Ending node**	**Part of biggest component in N imputed datasets**
L caudal middle frontal gyrus	L rostral middle frontal gyrus	10/10
L frontal pole	L caudate	10/10
L frontal pole	L nucleus accumbens	10/10
L insula	L putamen	10/10
L lateral occipital gyrus	L insula	10/10
L lateral orbitofrontal cortex	L putamen	10/10
L lateral orbitofrontal cortex	L caudate	10/10
L lingual	L insula	10/10
L pars orbitalis	L putamen	10/10
L pars orbitalis	L superior temporal gyrus	9/10
L postcentral gyrus	L insula	10/10
L precentral gyrus	L putamen	10/10
L precentral gyrus	L thalamus	8/10
L rostral middle frontal gyrus	L superior temporal gyrus	10/10
L rostral middle frontal gyrus	L insula	8/10
L superior frontal gyrus	L frontal pole	9/10
L superior frontal gyrus	R caudal anterior cingulate	9/10

A network within the right hemisphere containing 12 connections and 10 brain areas was close to significance (*p* = 0.059).

## 4 Discussion

In the present study, we identified a structural brain network that relates to language at school age in extremely preterm-born children. This network comprised 16 brain regions in the left hemisphere and one region in the right hemisphere. Within this network, higher FA values related to higher language scores. Neonatal brain injury was not associated with language outcome at school age. These findings suggest that extremely preterm children rely mostly on their left hemisphere during language processing, which is similar to typically developing children. However, they seem to use compensatory neural pathways that include brain areas right next to the areas typically involved in language processing. These areas include the pars orbitalis (adjacent to Broca's area) and the putamen and caudate nucleus (adjacent to the limbic system). Language difficulties were related to SES, but not to brain injury around birth.

### 4.1 Language scores

In our sample of EPT children, 22 % had a norm score below average on the language task. In a typical population it would be expected that 16 % fall in this range (Semel et al., 2010). This result is similar to previous studies, which report oral language problems in approximately 20–45 % of school-aged preterm children (e.g. Luu et al., 2009, Reidy et al., 2013, Stipdonk et al., 2020) and implies that it is clinically relevant to follow-up on language functions in these children.

Regarding potential risk factors for language outcome, only socioeconomic status (SES), measured by maternal education level, was related to language scores. That is, children from a higher SES background performed significantly better on the language task than children from a lower SES background. SES is a common predictor of language development in typically developing children (Pace et al., 2017). In preterm children previous studies show contradicting results. A study by Landry et al showed that SES was predictive for language scores in preterm children at school age (Landry et al., 2002). In contrast, in a previous *meta*-analysis, preterm children had lower scores on complex language functions independent from SES (Van Noort-Van der Spek et al., 2012). Furthermore, in a recent study by Stipdonk et al. (2020) maternal education level did not predict complex language scores in very preterm born children at school-age. However, the level of maternal education in their sample was not normally distributed, whereas in our sample the groups of low/middle and high SES were roughly equal (28 vs 30 participants, respectively).

Surprisingly, brain injury at term-equivalent age assessed using the Kidokoro score was not related to language scores in our sample. In another study, looking solely at white matter abnormalities, it was found that white matter injury was predictive of semantics, grammar, discourse, and phonological awareness in preterm school-aged children (Reidy et al., 2013). It should be noted that in our sample relatively few children had brain abnormalities, with a vast majority (87 %) scoring in the normal or mild abnormal range. In accordance, other studies found that even preterm children without severe neonatal brain injury had impaired language functions and increased needs for language-related educational resources compared to term children (Luu et al., 2009). Potentially, language problems in the absence of major brain injury can be explained by microstructural abnormalities in the brain because of preterm birth such as decreased FA in tracts subserving language functions (Constable et al., 2008, Vohr, 2014). However, also other factors such as environmental factors (i.e. educational environment) related to SES can play a role.

### 4.2 Structural brain language network in EPT children

In the present study, we found an interconnected network of white matter tracts in which FA values were related to performance on a complex language task. These white matter tracts connect brain areas in the left hemisphere, including the caudate, pre-and postcentral gyrus, caudal and rostral middle frontal gyrus, superior frontal gyrus, frontal pole, insula, lingual, nucleus accumbens, lateral occipital gyrus, lateral orbitofrontal cortex, pars orbitalis, putamen, superior temporal gyrus, and the thalamus. Additionally, the caudal anterior cingulate cortex in the right hemisphere was part of the language network in our EPT sample.

#### 4.2.1 Comparison to the dual-stream model in typically developing children

In the language network of EPT children, we found white matter connections that might correspond to the dorsal and ventral pathways of the dual-stream model (Hickok and Poeppel, 2004, Hickok and Poeppel, 2007). In adults and typically developing children, the dorsal pathways for language processing consist of the superior longitudinal fasciculus (SLF) and the arcuate fasciculus (AF). The main stream of the ventral route is the inferior fronto-occipital fasciculus (IFOF). In addition, the uncinate fasciculus (UF), the extreme capsule fiber system (ECFS), the middle longitudinal fasciculus (MLF) and the inferior longitudinal fasciculus (ILF) have been related to the ventral route. The language network identified in EPT children included a connection between the rostral middle frontal gyrus (MFG) and the superior temporal gyrus (STG). Tractography analysis showed that the MFG and the STG are connected by both the AF and the UF (from the dorsal and ventral streams) (Briggs et al., 2021, Hazem et al., 2021). The STG is well-known to be involved in language comprehension and recent studies have also shown a role for the MFG within the language network, next to its involvement in reorienting attention and working memory (Fox et al., 2006, Hazem et al., 2021, Rypma et al., 1999). The MFG has been related to both phonological and semantic language functions, and thus playing a role in both the dorsal and ventral language streams (Briggs et al., 2021, Hazem et al., 2021).

Another connection from our network that fits with the dual-stream model is the pathway we found between the pars orbitalis (parsOr), located in the inferior frontal gyrus (IFG) adjacent to Broca’s area, and the superior temporal gyrus (STG). A previous tractography study found that the UF connects the parsOr with the STG (Briggs et al., 2021). This pathway might also correspond to the AF, which projects from the STG to another brain structure within the IFG, namely the pars opercularis (Axer et al., 2013; E. F. Chang et al., 2015). Activation of the parsOr has been related to semantic retrieval (Price, 2010), a function proposed to include ventral pathways.

Finally, we found a connection between the lingual gyrus and the insula, which might correspond to a part of the IFOF. That is, the IFOF originates in the lingual gyrus and cuneus in the occipital lobe, as well as in parts of the temporal lobe, and then travels anteriorly to the inferior lobe, adjacent to the insula (Conner et al., 2018).

#### 4.2.2 Differences between the EPT language network and the dual-stream model

The EPT language network that we identified in the present study, also included connections beyond the dorsal and ventral pathways. First, there were many pathways involving the basal ganglia. These pathways connected the caudate, nucleus accumbens, and putamen to frontal lobe regions such as the frontal pole, insula, lateral orbitofrontal cortex, pars orbitalis, and precentral gyrus. The basal ganglia has previously been identified as playing a role in language processing in adults (Booth et al., 2007).

Other studies taking a whole-brain approach also found associations beyond the dorsal and ventral pathways with language in preterm children. They found the corpus callosum (CC), forceps (major and minor), thalamic radiation, cortico-spinal tract, and cerebellum to be involved (Feldman et al., 2012, Mürner-Lavanchy et al., 2018).

Finally, contrary to the dual stream model, the EPT language network did not comprise connections corresponding to the SLF, a fronto-parietal tract within the dorsal stream.

We speculate that EPT children might use compensatory neural pathways for language to make up for white matter injury of prematurity (Barnes-Davis et al., 2020, Kwon et al., 2016). Most areas involved in the network are also involved in higher-order cognitive functions. Performing tasks often necessitate substantially higher cognitive resources in preterm children, thereby resulting in a greater cognitive load compared to term-born children during testing. However it might also be due to the fact that our language task (sentence repetition) was more complex than in other studies. Regarding lateralization of language, it is known that children with atypical language development (such as with a developmental language disorder) show less lateralization in language organization (Verly et al., 2019). We also found a network in the right hemisphere that was close to significance, suggesting less lateralization.

The present study was limited by the fact that we only had good-quality MRI and language assessment in 58 out of 95 children. In addition, in 9 children the language task was terminated too early. Since this was due to an administration error by the tester and not related to child characteristics we do not expect this to generate bias. We tried to overcome this by using an imputation model. Furthermore, we did not have a large variability in SES. The generalizability of our result to this population needs further investigation. The major strength of this study is that we used a data-driven approach and therefore were not limited by region of interest analyses on language areas previously identified in typically developing children.

Since preterm children might recruit different neural pathways to compensate for altered brain development, it is important to not only focus on neural pathways already known to play a role in language in typically developing children and adults. In addition, preterm infants with lower SES (but not necessarily with a high Kidokoro score) warrant closer follow-up of language functions throughout childhood.

## 5 Conclusion

In preterm-born children, a structural brain network was identified that relates to complex language at school age, comprising 16 regions in the left and one in the right hemisphere. This network consists of areas from the dorsal and ventral pathways associated with language but also other, novel brain regions. Our study found no correlation between brain injury, assessed using the Kidokoro score, and language abilities. On the other hand, language scores were related to SES. EPT children might use compensatory neural pathways for language to make up for altered brain development and neuronal dysmaturation after preterm birth.

## CRediT authorship contribution statement

**M. Boumeester:** Writing – original draft, Validation, Methodology, Investigation, Formal analysis. **E. Blom:** Writing – review & editing, Supervision, Methodology, Investigation, Funding acquisition, Conceptualization. **T. Boerma:** Writing – review & editing, Validation, Supervision, Methodology, Investigation, Formal analysis, Conceptualization. **F. Lammertink:** Writing – review & editing, Methodology, Formal analysis, Data curation. **M.P. van den Heuvel:** Writing – review & editing, Supervision, Methodology, Formal analysis, Conceptualization. **J. Dudink:** Writing – review & editing, Validation, Supervision, Resources, Methodology, Data curation, Conceptualization. **M.J.N.L. Benders:** Writing – review & editing, Supervision, Methodology, Investigation. **E. Roze:** Writing – review & editing, Writing – original draft, Visualization, Validation, Supervision, Project administration, Methodology, Investigation, Funding acquisition, Formal analysis.

## Declaration of Competing Interest

The authors declare that they have no known competing financial interests or personal relationships that could have appeared to influence the work reported in this paper.

## Data Availability

Data will be made available on request.
