# Supplementary material for: Structural brain network in relation to language in school-aged extremely preterm children: A diffusion tensor imaging study
Source: Neuroimage Clin. 2025 Apr 12;46:103782. doi: 10.1016/j.nicl.2025.103782 (PMC12051154; doi:10.1016/j.nicl.2025.103782)
Supplement: Supplementary Data 1 [file mmc1.pdf]

Script 1

```r
# Open R and load the packages mice and lattice

setwd()

##PERFORMING IMPUTATIONS

#load dataset
dataset <- read.csv2()

options("install.lock"=FALSE)
install.packages("mice")
require(mice)
require(lattice)
set.seed(123)
install.packages("psych")
require ("psych")

summary(dataset) # summary of data set
md.pattern(dataset) #check missingness pattern

#create pred-matrix according to correlations e.g. r=.3
ini <- mice(dataset, pred=quickpred(dataset, mincor=.3), print=F)
pred <- ini$pred

# Multiple imputation
imp <- mice(dataset, pred = pred, m = 10, seed = 123, print=F)
imp$imp #shows the imputations for each 'round'
plot(imp)
# m=10 is the number of imputed data sets

# Explore convergence imputed data
imp40 <- mice(dataset, pred = pred, maxit = 35, seed = 123, print=F)
plot(imp40) #Check if there is no trend
plot(imp40, c()) #if you want to check 1 variable

#Export imputed datasets to SPSS
mids2spss(
  imp,
  filename = "[FILENAME]",
  path = getwd(),
  compress = FALSE,
  silent = FALSE
)

## POOLING P-VALUES NBS
m <- 10
p <- c() #insert 10 p-values
```

```
p <- round(p, 3)
Z <- qnorm(1 - p)      # z-transformation
Zbar <- mean(Z)
Ubar <- 1
Bm <- var(Z)           # between imputation variance
Tm <- Ubar+Bm*(1+1/m)  # total variance
rm <- (1+1/m)*Bm/Ubar  # fraction of missing information due to nonresponse
nu <- (m-1)*(1+1/rm)^2 # degrees of freedom for t-distribution
1 - pt(Zbar / sqrt(Tm), df = nu) #pooled p-value

#POOLING SD'S OF IMPUTED NORM SCORES
#Pooled SD: MeanLogSD <- (log(SD1) + log(SD2) + log(SDk))/k
#           exp(MeanLogSD)
LogMeanSD <-(log(2.889) + log(2.796) + log(2.985) + log(2.796) + log(2.875) +
log(2.796) + log(2.994) + log(2.796) + log(2.959) + log(2.796) + log(3.063) +
log(2.796) + log(3.006)+ log(2.817) + log(2.981) + log(2.796) + log(2.999) +
log(2.796) + log(3.024) + log(2.796))/20
exp(LogMeanSD)

#POOLING T-TESTS AND CORRELATIONS
#multilingualism
pool.scalar(Q = c(.054, -.098, -.057, -.229, -.188, -.007, -.492, -.209, -
.340, -.249), U = c(1.072^2, 1.094^2, 1.070^2, 1.095^2, 1.090^2, 1.110^2,
1.096^2, 1.094^2, 1.097^2, 1.102^2), n = 58)
#gender
pool.scalar(Q = c(.561, .588, .460, .588, .679, .424, .588, .561, .707, .543),
U = c(.871^2, .812^2, .873^2, .814^2, .808^2, .898^2, .883^2, .880^2, .814^2,
.888^2), n = 58)
#handedness
pool.scalar(Q = c(-.121, -.429, -.228, -.512, -.478, -.463, -.429, -.537, -
.537, -.571), U = c(.852^2, .868^2, .851^2, .869^2, .865^2, .881^2, .872^2,
.867^2, .871^2, .873^2), n = 58)
#SES
pool.scalar(Q = c(-1.976, -1.805, -1.940, -1.736, -1.802, -1.738, -1.667, -
1.700, -1.700, -1.702), U = c(.731^2, .755^2, .731^2, .759^2, .752^2, .770^2,
.764^2, .759^2, .763^2, .765^2), n = 58)
# CELF language test at hospital vs home
pool.scalar(Q = c(1.571, 1.545, 1.597, 1.545, 1.597, 1.492, 1.468, 1.571,
1.571, 1.518), U = c(.789^2, .807^2, .787^2, .809^2, .802^2, .822^2, .813^2,
.807^2, .810^2, .815^2), n = 58)
#brain abnormality
fisherz(-.167) #correlation coefficients need to be z-transformed first,
.5(ln(1+r) / ln(1-r))
1/sqrt(55-3) #Standard error, 1/sqrt(n-3)
pool.scalar(Q = c(-0.1799165, -0.1757886, -0.149095, -0.1757886, -0.1613867, -
0.1460292, -0.1799165, -0.1624131, -0.1809494, -0.168579), U = c(0.138675^2,
0.138675^2, 0.138675^2, 0.138675^2, 0.138675^2, 0.138675^2, 0.138675^2,
0.138675^2, 0.138675^2, 0.138675^2), n = 55)
fisherz2r(-0.1679863) # transform back to correlation coefficient
```

```
#Calculate P-value
statistic <- 1.5475 / sqrt(0.6519634) # qbar / sqrt(t)
p.value <- 2 * (1 - pt(abs(statistic), pmax(54.92271, 0.001))) #pmax(df,
0.001)
p.value
statistic #is t-value t-test

#cohen's d
#(M2 - M1)/SDpooled
(8.90-7.36)/2.886159
```
