# Supplementary material for: Structural brain network in relation to language in school-aged extremely preterm children: A diffusion tensor imaging study
Source: Neuroimage Clin. 2025 Apr 12;46:103782. doi: 10.1016/j.nicl.2025.103782 (PMC12051154; doi:10.1016/j.nicl.2025.103782)
Supplement: Supplementary Data 2 [file mmc2.pdf]

Script 2

```
################################ BIOS: NBS language
##############################
## setwd
setwd()

## packages
options("install.lock"=FALSE)
install.packages("R.matlab")
library(R.matlab)
install.packages('devtools')
library(devtools)
devtools::install_github('cwatson/brainGraph')
library(brainGraph)
install.packages('data.table')
library("data.table")
install.packages("biclust")
library(biclust)
install.packages("Matrix")
library(Matrix)
install.packages("doMC")
library(doMC)
install.packages("pacman")
library(pacman)
install.packages("snow")
install.packages("doSNOW")
install.packages("ggpubr")
library(ggpubr)

## Multicore processing
OS <- .Platform$OS.type
if (OS == 'windows') {
  library(snow)
  library(doSNOW)
  num.cores <- as.numeric(Sys.getenv('NUMBER_OF_PROCESSORS'))
  cl <- makeCluster(num.cores, type='SOCK')
  clusterExport(cl, 'sim.rand.graph.par')   # Or whatever functions you will
use
  registerDoSNOW(cl)
} else {
  library(doMC)
  registerDoMC(detectCores() - 1L)  # Keep 1 core free
}

pacman::p_load(brainGraph, doMC)
registerDoMC(detectCores())

## Subset function (run once)
```

```r
subset_graph <- function(g, condition) {
  stopifnot(nzchar(condition))

  # Function for creating the condition string to later subset the graph
  get_cond_string <- function(orig) {
    spec <- '\\s\\&\\s|\\s\\|\\s'  # Splits are either " & " or " | "
    conditions <- strsplit(orig, split=spec)[[1L]]
    if (length(conditions) > 1L) {  # Multiple conditions
      if (isFALSE(grepl(spec, orig))) {
        stop('Logical operators must be surrounded by spaces!')
      }
      nchars <- cumsum(nchar(conditions))
      endpts <- nchars + seq.int(from=2L, by=3L, length.out=length(nchars))
      splits <- vapply(endpts, function(x) substr(orig, start=x, stop=x),
character(1L))
      conditions <- trimws(conditions) # Remove unnecessary whitespace

      cond.string <- paste(vapply(seq_along(conditions), function(x)
        paste0('V(g)$', conditions[x], splits[x]), character(1L)),
        collapse='')
    } else {
      cond.string <- paste0('V(g)$', conditions)
    }
    return(cond.string)
  }

  # Handle when logical expressions are separated by parentheses
  if (isTRUE(grepl('\\(.*\\&.*\\)', condition)) ||
isTRUE(grepl('\\(.*\\|.*\\)', condition))) {
    subs <- strsplit(condition, split='\\) & \\(')[[1L]]
    subs <- as.list(trimws(subs, whitespace='[\\(\\) ]'))
    cond.strings <- vapply(subs, get_cond_string, character(1L))
    cond.string <- paste0('(', paste(cond.strings, collapse=') & ('), ')')
  } else {
    cond.string <- get_cond_string(condition)
  }

  cond <- eval(parse(text=cond.string))
  if (sum(cond, na.rm=TRUE) == 0L) {
    warning('No vertices meet criteria! No graph created')
    g <- inds <- NULL
  } else {
    inds <- which(cond)
    cond <- setdiff(seq_len(vcount(g)), inds)
    orig.class <- class(g)
    g <- delete.vertices(g, cond)
    class(g) <- orig.class
  }
  list(g=g, inds=inds)
```

```r
}

## Load demographics
premCov <- read.csv2()
colnames(premCov)[1] <- "SubjectID"
## Specify missing value
premCov[premCov==999] <- NA

## ONLY IF RELEVANT: Remove subject with missing data
premCov <- premCov[!is.na(premCov$...),]

## Combine age parameter
premCov$Age_scan <- premCov$Age_Scan_years + (premCov$Age_Scan_months / 12)
premCov$Age_CELF <- premCov$Age_CELF_years + (premCov$Age_CELF_months / 12)
premCov$GA <- premCov$GA_weeks + (premCov$GA_days / 7)

## Difference in assessment time CELF-MRI
premCov$DiffTime <- premCov$Age_CELF - premCov$Age_scan
summary(premCov$DiffTime)

## load matrices
connectivity <- readMat()
str(connectivity)
dim(connectivity$connectivity)[1L:4L] #82 82  14  73

## Check similarity atlas and connectivity matrix: should reorder!
dk.scgm
as.data.frame(unlist(connectivity$regionDescriptions))
write.csv2(dk.scgm, "DK_atlas.csv", row.names=FALSE)

## Original atlas order
custom_atlas_orig <- read.csv2("DK_atlasC.csv")

## Custom atlas; reorder own atlas so it matches dk.scgm
custom_atlas <- read.csv2()
setDT(custom_atlas)
custom_atlas <- as_atlas(custom_atlas)

## Remove outliers: not part of language sample
# {'sub-BIOS005'}
# {'sub-BIOS040'}
# {'sub-BIOS053'}

SubjectstoRemove <- as.data.frame(unlist(connectivity$subjects))
colnames(SubjectstoRemove)[1] <- "SubjectID"
SubjectstoRemove$SubjectID <- gsub("sub-", "", SubjectstoRemove$SubjectID)
which(SubjectstoRemove$SubjectID %in% premCov$SubjectID)
```

```
connectivity$connectivity <-
connectivity$connectivity[,,,c(which(SubjectstoRemove$SubjectID %in%
premCov$SubjectID))]
connectivity$subjects <- as.data.frame(unlist(connectivity$subjects))
connectivity$subjects <-
connectivity$subjects[c(which(SubjectstoRemove$SubjectID %in%
premCov$SubjectID)),]

setdiff(connectivity$subjects, paste0("sub-", premCov$SubjectID))

## Select correct connectivity file
mat <- connectivity
mat$connectivity <- mat$connectivity[,,3,] #select FA
dim(mat$connectivity)[3L] #58 subjects

## Reorder connectivity matrices based on index (matching dk.scgm)
mat$connectivity_reorder <- mat$connectivity
for (i in 1:nrow(premCov)) {
  temp <- as.matrix(mat$connectivity[,,i])
  colnames(temp) <- rownames(temp) <- custom_atlas_orig$name
  mat$connectivity_reorder[,,i] <-
temp[custom_atlas$index,custom_atlas$index,drop=FALSE]
}

## Check if values do not vary
## Orig
orig <- as.matrix(mat$connectivity[,,6])
colnames(orig) <- rownames(orig) <- custom_atlas_orig$name
orig['lSFG', 'rMOF'] #0.4808537

## Reord
reord <- as.matrix(mat$connectivity[,,6])
colnames(reord) <- rownames(reord) <- custom_atlas_orig$name
reord <- reord[custom_atlas$index,custom_atlas$index,drop=FALSE]
reord['lSFG', 'rMOF'] #0.4808537

## Calc mean FA per individual
MeanFA <- NULL
for (i in 1:nrow(premCov)) {
  MeanFA[i] <- sum(mat$connectivity_reorder[,,i]) /
Matrix::nnzero(mat$connectivity_reorder[,,i], na.counted=FALSE)
}
premCov$meanFA <- MeanFA

## Control for prevalence edges, activate biclust package
my.matsBin <- binarize(mat$connectivity_reorder, threshold=0)
prev <- rowSums(my.matsBin, dims =2) / dim(mat$connectivity_reorder)[3L]

my.matsPrev <- NULL
```

```
my.matsPrev$A <- mat$connectivity_reorder

## prevalence threshold: adjust accordingly
my.matsPrev$A[prev < .90] <-0

## Running NBS: Changing covariates at lines 193 and 204 (make sure to adjust
the contrast [at line 197] as well)
## Current covariates: gender (2), SES_mother(18) CELF_norm (36 etc.),
Age_scan (68), AgeDifferenceCELFscan (69)
str(premCov)
setDT(premCov)
DesignMatrix <- brainGraph_GLM_design(premCov[, c(2, 18, 39, 68, 69)],
factorize=c("Gender", "SES_mother"), coding='effects') #center.mean /
center.by
rownames(DesignMatrix) <- premCov$SubjectID

head(DesignMatrix)
con.mat <-list(matrix(c(0, 0, 0, 1, 0, 0), nrow=1,
dimnames=list('CELF_norm_2')))

## Error in apply(x, 3L, qr.default, ...) : 'MARGIN' does not match dim(X):
due to R > 4.0.0, below a snippet that solves this issue
dim(DesignMatrix)[2] == qr(DesignMatrix)$rank #TRUE
qr.matrix <- function(x, ...) {
  qr.default(x, ...)
}
res.nbs <- NBS(my.matsPrev$A, premCov[, c(2, 18, 39, 68, 69)], con.mat,
               X=DesignMatrix, p.init=0.05, N=10e3,
con.type='f',alternative='two.sided', long=TRUE) #does not take the custom
atlas

summary(res.nbs)
nbs.dt <- with(res.nbs, data.table(alt=alt, N=N, components$observed,
ecount=0))

##EXAMPLE (prev. threshold 50%):
#      contrast # vertices # edges p-value
# [1,]        1         15      14 0.006199 **
# [2,]        1          9       8 0.056694 .
# [3,]        1          3       2 0.922608
# [4,]        1          2       1 0.999500
# [5,]        1          2       1 0.999500
# [6,]        1          2       1 0.999500
# [7,]        1          2       1 0.999500
# [8,]        1          2       1 0.999500
# [9,]        1          2       1 0.999500

## Find edges/regions involved
#COMPONENT 1
```

```
g.nbs <- make_brainGraphList(res.nbs, "dk.scgm", set.attrs=FALSE,
.progress=FALSE, modality = "dti") #if not atlas specified, default to
guess_atlas(res.nbs$T.mat) = "dk.scgm"
vcount(subset_graph(g.nbs[1L], paste('comp ==', 1))$g) #15
V(subset_graph(g.nbs[1L], paste('comp ==', 1))$g)

as_edgelist(subset_graph(g.nbs[1L], paste('comp ==', 1))$g)

#COMPONENT 2
g.nbs <- make_brainGraphList(res.nbs, "dk.scgm", set.attrs=FALSE,
.progress=FALSE, modality = "dti") #if not atlas specified, default to
guess_atlas(res.nbs$T.mat) = "dk.scgm"
vcount(subset_graph(g.nbs[1L], paste('comp ==', 2))$g) #15
V(subset_graph(g.nbs[1L], paste('comp ==', 2))$g)

as_edgelist(subset_graph(g.nbs[1L], paste('comp ==', 2))$g)
#EXAMPLE:
#      [,1]    [,2]
#[1,] "lIPL"  "lINS"
#[2,] "lLOG"  "lINS"
#[3,] "lLOF"  "lCAUD"
#[4,] "lLING" "lINS"
#[5,] "lpreC" "lTHAL"
#[6,] "lpreC" "lPUT"
#[7,] "lrMFG" "lACCU"
#[8,] "lSFG"  "lFP"
#[9,] "lSFG"  "lTHAL"
#[10,] "lSFG"  "rcACC"
#[11,] "lSFG"  "rMOF"
#[12,] "lFP"   "lCAUD"
#[13,] "lFP"   "lACCU"
#[14,] "lINS"  "lPUT"

nbs_vertices <- custom_atlas[as.character(custom_atlas$name) %in%
as_edgelist(subset_graph(g.nbs[1L], paste('comp ==', 1))$g),]
#see nbs_vertices in 'Environment' --> click on table icon
#save table nbs_vertices to .txt file
write.table(nbs_vertices,"[NAME].txt",sep="\t",row.names=FALSE)

res.nbs.Heatmap <- res.nbs$T.mat
rownames(res.nbs.Heatmap) <- unlist(dk.scgm$name)
colnames(res.nbs.Heatmap) <- unlist(dk.scgm$name)
levelplot(res.nbs.Heatmap, col.regions = rev(heat.colors(100)),
          main = "All F-values", ylab = "ROI", xlab = "ROI",
scales=list(x=list(cex=.5, rot = 90),y=list(cex=.5)))

## Keep edges involved in the significant component (edge list)
nnodes <- 82
avg_mx <- matrix(0, nrow = nnodes, ncol = nnodes)
```

```
edge_mat <- array(0, dim(avg_mx))
#edge_mat <- binarize(res.nbs$T.mat, threshold=0)
rownames(edge_mat) <- unlist(dk.scgm$name)
colnames(edge_mat) <- unlist(dk.scgm$name)

edge_mat[as_edgelist(subset_graph(g.nbs[1L], paste('comp ==', 1))$g, names =
TRUE)] <- 1

levelplot(edge_mat, col.regions =
rev(heat.colors(100)),scales=list(x=list(cex=.5, rot = 90),y=list(cex=.5)),
          main = "Component 1 p < 0.05", ylab = "ROI", xlab = "ROI")

## Calc meanFA of sub-network NBS: component 1
my.matsTemp <- my.matsPrev$A
my.matsTemp[edge_mat == 0] <- NA
for (i in 1:nrow(premCov)){
  premCov$ConnFA[i] <- sum(my.matsTemp[,,i], na.rm = TRUE) /
Matrix::nnzero(my.matsTemp[,,i], na.counted=FALSE)
}

## Association
ggplot(data = premCov, mapping = aes(x = CELF_norm, y = ConnFA)) +
  geom_point(size = 3, colour = "orange") + scale_x_log10() +
  geom_smooth(method = "lm", size = 1.5) + stat_cor(method="pearson") +
theme_bw() + xlab("Sentence repetition norm score") +
  ylab("Mean connectivity")

options(error=NULL)
plot(g.nbs[1], alpha=0.05,vertex.color=rep('orange', 15), vertex.lable = TRUE,
vertex.size = 8, edge.color=rep('black',14), edge.width = 2, mni=TRUE, plane =
'axial', cex = 1.25, main = "NBS results: Subnetwork related to language
task")

# Make sure that the colors have the same length as the significant component
(15 vertices, 14 edges)

# make a braingraph plot after imputations
edgelist <- matrix( c("lcMFG","lrMFG" , "lLOF","lCAUD" , "lrMFG","lACCU" ,
"lSFG","rcACC" , "lFP","lCAUD" , "lFP","lACCU" , "lSFG","lFP" , "lrMFG","lSTG"
, "lLOF","lFP" , "lLOG","lINS" , "lLING","lINS" , "lpostC","lINS" ,
"lPREC","lTHAL" , "lPREC","lPUT" , "lSFG","lTHAL" , "lINS","lPUT"), nc = 2,
byrow = TRUE)
graph <- graph_from_edgelist(edgelist, directed = TRUE)
braingraph <- make_brainGraph(graph, "dk.scgm", set.attrs=TRUE, modality =
"dti")
plot(braingraph, vertex.color=rep('red', 16), edge.color=rep('black',16),
line=5, cex=3, mni=TRUE, plane = 'axial', vertex.size=5) #gives an error, will
solve later!
```

```
#################################################################### END
```
